# The Use of Quality Benchmarking in Assessing Web Resources for the Dermatology Virtual Branch Library of the National electronic Library for Health (NeLH)

**DOI:** 10.2196/jmir.3.1.e5

**Published:** 2001-03-17

**Authors:** MN Kamel Boulos, AV Roudsari, C Gordon, JA Muir Gray

**Affiliations:** ^1^Centre for Measurement and Information in MedicineSchool of InformaticsCity UniversityLondonUK; ^2^Royal Brompton & Harefield NHS TrustLondonUK; ^3^Institute of Health SciencesUniversity of OxfordOxfordUK

**Keywords:** Internet, Quality of Health Care, Ethics, Dermatology, Libraries

## Abstract

**Background:**

In 1998, the U.K. National Health Service Information for Health Strategy proposed the implementation of a National electronic Library for Health to provide clinicians, healthcare managers and planners, patients and the public with easy, round the clock access to high quality, up-to-date electronic information on health and healthcare. The Virtual Branch Libraries are among the most important components of the National electronic Library for Health . They aim at creating online knowledge based communities, each concerned with some specific clinical and other health-related topics.

**Objectives:**

This study is about the envisaged Dermatology Virtual Branch Libraries of the National electronic Library for Health . It aims at selecting suitable dermatology Web resources for inclusion in the forthcoming Virtual Branch Libraries after establishing preliminary quality benchmarking rules for this task. Psoriasis, being a common dermatological condition, has been chosen as a starting point.

**Methods:**

Because quality is a principal concern of the National electronic Library for Health, the study includes a review of the major quality benchmarking systems available today for assessing health-related Web sites. The methodology of developing a quality benchmarking system has been also reviewed. Aided by metasearch Web tools, candidate resources were hand-selected in light of the reviewed benchmarking systems and specific criteria set by the authors.

**Results:**

Over 90 professional and patient-oriented Web resources on psoriasis and dermatology in general are suggested for inclusion in the forthcoming Dermatology Virtual Branch Libraries. The idea of an all-in knowledge-hallmarking instrument for the National electronic Library for Health is also proposed based on the reviewed quality benchmarking systems.

**Conclusions:**

Skilled, methodical, organized human reviewing, selection and filtering based on well-defined quality appraisal criteria seems likely to be the key ingredient in the envisaged National electronic Library for Health service. Furthermore, by promoting the application of agreed quality guidelines and codes of ethics by all health information providers and not just within the National electronic Library for Health, the overall quality of the Web will improve with time and the Web will ultimately become a reliable and integral part of the care space.

## Introduction

The U.K. National electronic Library for Health (NeLH) [1] is a Web-based, "library without walls" project announced in 1998 as part of the National Health Service (NHS) "Information for Health" strategy of the U.K. Department of Health [2]. It will be one of the cornerstones of the new NHS health information system, with the ultimate goal of giving the people of the United Kingdom the best healthcare service in the world.

Backed by specialized and supporting services like the National Institute for Clinical Excellence (NICE) and other evidence and quality benchmarking and assurance systems, the NeLH will provide easy, 24-hour access to the best current knowledge and consequently help improve health and healthcare, clinical practice and patient choice [3].

The NeLH will also reduce the variations in healthcare delivery and quality from one part of the United Kingdom to another and empower the public and patients, reducing inequalities and ending "knowledge poverty" (in access and quality). Furthermore, the NeLH will help its users cope with the "knowledge overload", often of poor or uncertain quality, coming from raw sources like the World Wide Web and even from specialized services like MEDLINE.

The NeLH will include vortals known as Virtual Branch Libraries (VBLs). A VBL is a special collection of resources relevant to a specific community (or more than one community) of users and related to their particular interests and needs [4]. A Dermatology VBL is among the planned VBLs and will be based on the same high quality principles as the rest of the NeLH. Psoriasis, a common dermatological condition, has been chosen as a starting point.

### Quality Benchmarking of Health-Related Web Resources

The Internet has become a major source of health information. No wonder the American Medical Informatics Association has a special Internet Working Group and a new peer-reviewed Journal of Medical Internet Research (JMIR) [5] was launched in 1999.

The importance of the Internet for healthcare professionals cannot be overlooked. Eysenbach et al [6] redefine cybermedicine as a new academic specialty at the crossroads of medical informatics and public health (medicine in cyberspace where cyberspace denotes the Internet). Compared with telemedicine (which is primarily curative medicine), cybermedicine is primarily preventive medicine and has changed the traditional model of preventive medicine and patient/community health promotion. Cybermedicine features mass patient education and patient-to-patient exchanges of information for patient education and self-support.

**Figure 1:**
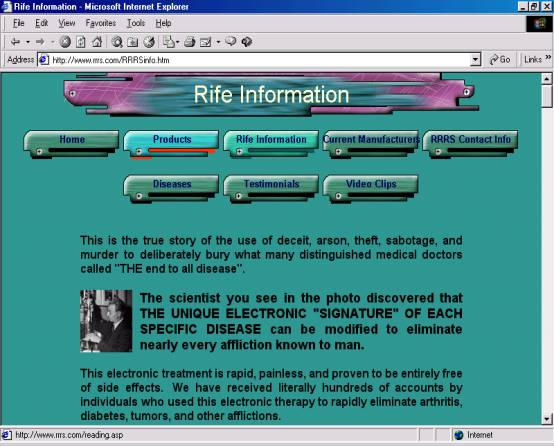
Royal Rife Research Society Web site, a blatant example of quackery on the Web [39].  The site offers a "miraculous" universal electronic cure for arthritis, diabetes, tumours and other afflictions (the end to all disease), and claims that the University of Southern California has sponsored research into this electronic therapy on the terminally ill, with astounding results

There is however a growing concern about the quality of health information on the Internet, which is frequently inaccurate or biased and sometimes even misleading and dangerous (Figure 1). Anyone can be a publisher on the Web, which is good for a democratic society but potentially problematic in professions such as medicine [7].

The NeLH will not act as a censor, but rather as a quality filter powered by an explicit quality measurement system. This role is analogous to that of the renowned Goldsmith's Company (founded in London in 1327), which never traded gold, but was able through its independent hallmark stamping system to set the standards for the quality and purity of gold being traded. Knowledge hallmarks are needed to perform the function of gold hallmarks. The Cochrane logo for example has become a knowledge hallmark for systematic reviews and readers can always refer to the Cochrane Collaboration Handbook [8] to see the methods used to appraise and produce the Cochrane Reviews [9].

## Methods

### A Review of the Major Quality Benchmarking Systems Available Today

Some of the systems discussed below are mainly codes of ethics for resource providers, e.g. HONcode [10], while others are true quality-rating tools, e.g. the Oxford Centre for Evidence-Based Medicine Levels of Evidence [11]. Both approaches are important and there have been few attempts to combine a code of best practice with true quality rating under a single umbrella. Furthermore, two of the systems reviewed below, namely HONcode and med-PICS/MedCERTAIN [12,13], require target resource modification if they are to function optimally, e.g. adding the HONcode "active seal" logo and code or adding some PICS-labels or metatags to the section of Web pages in case of med-PICS. To use med-PICS consumers must also load the med-PICS rating description (a '.rat' file) into their Web browsers. The other systems described below provide quality evaluation schemes or checklists, sometimes very sophisticated, for selection of quality resources (they do not impose any modifications on target resources), e.g. OMNI [14], DISCERN [15] and QUICK [16]. Some systems also offer a directory service for quality resources, e.g. HON, OMNI and Medical Matrix [17].

### Oxford Centre for Evidence-Based Medicine Levels of Evidence

Ball et al [11] from the Centre for Evidence-Based Medicine at Oxford define ten levels of evidence (1a, 1b, 1c, 2a, 2b, 2c, 3a, 3b, 4, 5) and map them to four grades of recommendation (A, B, C, D). A systematic review (with homogeneity) of randomized controlled trials is considered to be the most reliable source of evidence (top level), while an expert opinion (without explicit critical appraisal) is graded as the least trustworthy piece of evidence (level 5, Grade D). Systematic reviews act like a quality filter by purifying the findings of primary (raw) research, detecting bias and poor quality research and distilling the findings (the good reliable ones) into a single report.

### The Health on the Net Foundation Code of Conduct (HONcode)

An international initiative launched in early 1996, Health On the Net Foundation (HON) is a not-for-profit organization headquartered in Geneva, Switzerland. The major HON sponsors are Sun Microsystems, the Swiss Institute of Bioinformatics and the State of Geneva.

**Figure 2:**
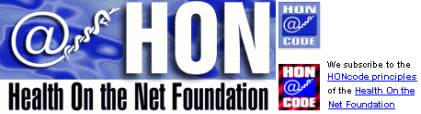
Health on the Net Foundation logo and HONcode blue and red seals

HON Code of Conduct (HONcode) [10] is a self-regulatory, voluntary certification system. HONcode does not rate the medical accuracy, validity or appropriateness of the information itself. It only defines a set of rules to hold Web site developers to basic ethical standards in the presentation of information and ensure readers always know the source and the purpose of the information they are reading. Compliant sites identify themselves by the blue-and-red HONcode hyperlink or "active" seal displayed usually at the bottom of their homepage (Figure 2).

HONcode addresses eight points: authority of the information provided, complementarity, confidentiality and privacy, proper attribution of sources, justifiability, transparency of authorship, transparency of financial sponsorship and honesty in advertising and editorial policy with emphasis on the importance of clearly separating advertising from editorial content. Dishonest operators may cut and paste the HONcode seal onto their Web sites. To check whether a given site featuring the HONcode seal is a bona fide HONcode subscriber, users can place their mouse cursor over the displayed HONcode seal and if the seal is authentic, they should see a special HONcode ID number appearing along the status bar of their browser. Clicking the seal should link them directly to a page on HON's site that summarizes the site's HONcode registration status. HON also has its own policing system and conducts random checks on subscribers.

### Internet Healthcare Coalition e-Health Code of Ethics

**Figure 3:**
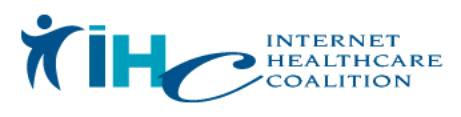
Internet Healthcare Coalition logo

The Internet Healthcare Coalition (IHC) (Figure 3) [18] is a not-for-profit organization with four major Internet health information providers, including Medscape, drkoop.com and Mediconsult, in addition to GlaxoSmithKline, the British pharmaceutical company, among its sponsors. (These sponsors should not pose any conflict of interest according to the Coalition's Statement of Independence).

In May 2000, the IHC e-Health Ethics Initiative introduced the Washington e-Health Code of Ethics which sets forth some important guiding principles for Internet healthcare sites and services grouped under eight main headings: candor; honesty; quality; informed consent; privacy; professionalism in online healthcare; responsible partnering; and accountability [19]. The full code can be downloaded from http://www.ihealthcoalition.org/ethics/code0524.pdf [accessed 27 August 2000].

### Health Internet Ethics Consortium

**Figure 4:**
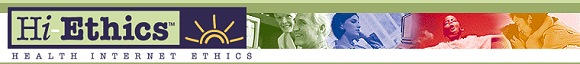
Hi-Ethics Consortium Logo

In May 2000 the Hi-Ethics Consortium (Figure 4) [20], a coalition that brings together some of the most widely used health Internet sites, published their thoughts on what constitutes good Internet ethics. The Hi Ethics Consortium summarizes the key ethical principles of electronic health information publishing on the Web into a 14-point list. The complete list of Principles and a useful glossary is available [20].

### MedCERTAIN (med-PICS Certification and Rating of Trustful and Assessed Health Information on the Net)

One way to ensure that only quality medical Web sites are delivered to the consumers is to configure their Web browsers to filter out information that does not meet a defined standard [21].

**Figure 5:**
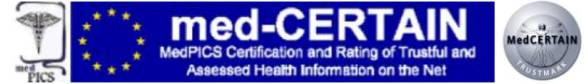
med-PICS and MedCERTAIN logos

MedCERTAIN (Figure 5) and the closely related metadata vocabulary med-PICS are initiatives for assessing, rating, labeling and filtering health information on the Web [12,13].

MedCERTAIN, an international not-for-profit project, was launched in 2000. Backed by the European Union under the "EU Action Plan for Safer Use of the Internet," it aims at improving health information quality on the Web through raising consumer awareness and industry self-governance. The underlying idea, proposed by G. Eysenbach, is to label medical Web pages with meta-information (information about information) using a standard computer-readable vocabulary based on the PICS (Platform for Internet Content Selection) labeling scheme [22].

These meta-information labels may be descriptive or evaluative. For example, a book review or critique is considered evaluative meta-information, while the table of contents is descriptive meta-information. Under the MedCERTAIN/med-PICS scheme, authors of medical Web pages are encouraged to include Unified Medical Language System (UMLS) codes in the header of their pages, as this can greatly enhance the quality of Web queries and increase the relevancy of search results. The proposed med-PICS vocabulary also includes definitions of target audience and country, type of information, contents rating, source assessment and advertising policy.

MedCERTAIN [13] will be a self- and third-party rating system. The project will develop and apply the necessary technologies to label health information on the Web, including exploring the next generation of PICS, which will apply Resource Description Framework (RDF), and eXtensible Markup Language (XML). A technical and organizational infrastructure will allow associations (e.g. medical societies) and individuals (e.g. medical domain experts) to rate (i.e. to assign meta-information to) health information on the Internet, in a collaborative, distributed and decentralized way.

Consumers will be able to use their browsers or additional software or search engines to retrieve this meta-information automatically in the background whenever they access a Web site. Based on this meta-information, access can be limited to quality assessed content on the Web and a disclaimer may be displayed if the consumer leaves the rated subset of the Web. The MedCERTAIN consortium will create different levels of certification for publishers of health information on the Web, ranging from simple quality seals indicating a "good standing" of the site (level 1) to quality seals indicating that the site has been peer-reviewed externally. Web sites that want to get a MedCERTAIN certification will have to commit themselves to the Washington Code of e-Health Ethics or other ethical codes (see above). A community of trusted assessors will rate information as they surf the Web, flagging fraudulent information and evaluating (peer-reviewing) information if authors apply for a "level-3" quality seal.

**Figure 6:**
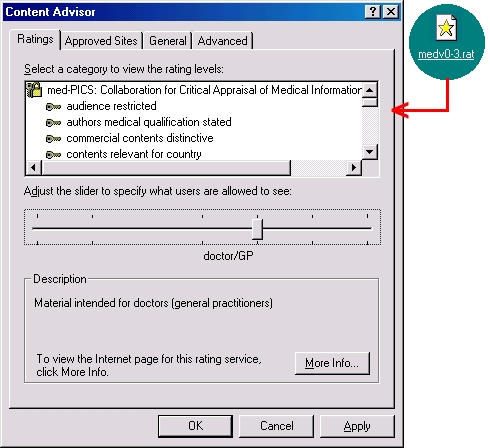
Defining the target audience in Microsoft Internet Explorer Content Advisor using med-PICS after loading the proposed med-PICS rating file (current version as of May 2000 is named 'medv0-3.rat'). In this screenshot, the target audience has been defined as a doctor/GP

To make use of med-PICS (Figure 6) [12], a rating description must be loaded into the Web browser. Microsoft Internet Explorer simply needs a text file, with the file extension '.rat.' Once loaded, the user is presented with a simple interface through which quality requirements and personal preferences can be defined (Figure 6). Users then need to define which labeling bureau(x) they wish to check with before accessing any Web site.

Subsequently, when a user requests, through the browser, any Web page, the software filter not only fetches the document but also makes an inquiry to the label bureau requesting any labels that have been assigned to that site. Depending on what the labels say, the filter may display an alert or disclaimer [21].

### OMNI (Organising Medical Networked Information)

Based at the University of Nottingham and backed by people from a wide array of backgrounds and institutions, including several other universities, the Department of Health, the Wellcome Trust and the British Library, OMNI (Figure 7), which started in 1994, is now a much-respected UK point of access to quality biomedical Internet resources [14]. OMNI has recently become one of BIOME's health and life sciences gateways [23].

OMNI's Advisory Group on Evaluation Criteria (AGEC) started by examining a number of different services available via the Internet that seek to provide access to selected and evaluated medical networked information resources. The group compared the criteria these services use for evaluating resources before producing their own OMNI Guidelines for Resource Evaluation which constitute one of the most comprehensive quality benchmarking checklists available today [24].

Resources are included that are either accessible directly as Web pages or can be downloaded across the network, e.g. downloadable tutoring software. As a rule, OMNI does not point to sites as a whole but to specific resources, so resources are identified and indexed at the level of individual Web pages and downloadable files (i.e., cataloguing on a per-page as opposed to per-site basis). This rule is clearly unsustainable in the case of databases, electronic journals and similar resources with a huge content base, where, unless an individual article or posting is particularly valuable in its own right and offers unique insights, OMNI will normally only point to the homepages of such resources.

**Figure 7:** OMNI logo

OMNI's selection process starts by establishing the context of the candidate resource and examining its scope, target audiences, authority and provenance. The evaluation criteria can be grouped into two main sets: content evaluation criteria and access evaluation criteria.

Content evaluation criteria include coverage, accuracy of information content, currency/frequency and regularity of updating and uniqueness/comparison with other sources. Access evaluation criteria include accessibility, usability and charging policy (if there are access restrictions), any special requirements, software reliability (for software resources), copyright and redistribution issues, language, design and layout/user interface and finally user support/documentation. Each criterion consists of a series of questions (checklist) to which the assessors are supposed to find answers.

The resource evaluation process considers all the criteria listed above, some of which might not be fully satisfied (or might not be applicable to the type of resource in question). In the final analysis, it is the overall impression about the value of a resource to the OMNI user community that guides the assessors to recommend it for inclusion in the OMNI database.

### DISCERN

**Figure 8:**
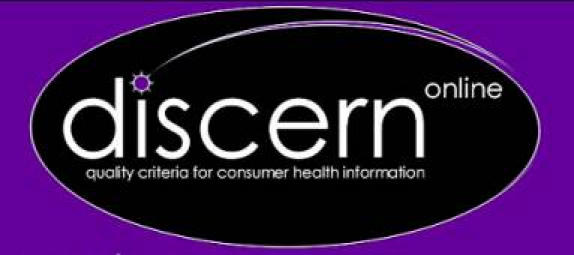
DISCERN logo

The DISCERN Project (Figure 8) [15] was funded by The British Library and the NHS Research & Development Program at the University of Oxford.

DISCERN is an instrument designed to help users of consumer health information judge the quality of written information about treatment choices. DISCERN is suitable for anyone who uses or produces information about treatment choices. To pass the DISCERN benchmark, a good quality publication about treatment choices must:

- have explicit aims;
- achieve its aims;
- be relevant to consumers;
- make sources of information explicit;
- make date of information explicit;
- be balanced and unbiased;
- list additional sources of information;
- refer to areas of uncertainty;
- describe how treatment works;
- describe the benefits of treatment;
- describe the risks of treatment;
- describe what would happen without treatment;
- describe the effects of treatment choices on overall quality of life;
- make it clear there may be more than one possible treatment choice;
- provide support for shared decision-making.

### C-H-i-Q (Centre for Health Information Quality - UK)

The Centre for Health Information Quality (C-H-i-Q) (Figure 9) was established in 1997 as part of the Patient Partnership Strategy, an NHS initiative acknowledging the need to "put patients first." C-H-i-Q has an international reputation, in particular in the area of appraisal of consumer health information and promoting good quality patient information [25]. C-H-i-Q has developed close ties with NICE to provide patients in the future with clear, concise "consumer-friendly" versions of NICE clinical guidelines.

**Figure 9:** C-H-i-Q logo

Another major responsibility of the Center is to provide an appraisal service for NHS Direct Online, the NHS patient information service [26]. The following themes are on C-H-i-Q's checklist for resource evaluation:

- accessibility (the information is in an appropriate format for the target audience);
- accuracy (the information is based on the best available evidence);
- appropriateness (the information communicates relevant messages);
- availability (the information is available to the widest possible audience);
- currency (the information is up-to-date);
- legibility (written information is clearly presented);
- originality (information has not already been produced for the same audience and in the same format);
- patient involvement (the information is specifically designed to meet the needs of the patient);
- readability (words and sentences are kept short where possible and jargon is minimized);
- reliability (the information addresses all essential issues).

### QUICK (The QUality Information ChecKlist)

**Figure 10:**
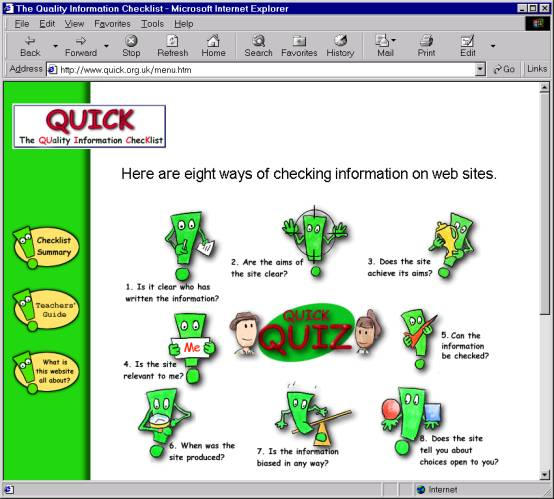
Screenshot from the QUality Information ChecKlist Web site showing the eight points consumers have to consider when assessing the quality of information on Web sites

The QUICK (QUality Information ChecKlist) Web site (Figure 10) [16] was developed by C-H-i-Q (Centre for Health Information Quality) in collaboration with HEA (the Health Education Authority (now replaced by the Health Development Agency, HDA). QUICK is a resource to help young people evaluate the information they find on the Internet. QUICK checklist includes the following eight questions:

1. Is it clear who has written the information? Who is the author? Is it an organization or an individual person? Is there a way to contact them? An e-mail address on its own is not a proof that the author is a genuine expert on a subject or even who they claim to be as anyone can get an e-mail address especially with free services like Hotmail.
2. Are the aims of the site clear? What are the aims of the site? What is it for? Who is it for?
3. Does the site achieve its aims? Does the site do what it says it will?
4. Is the site relevant to me?
5. Can the information be checked? Is the author qualified to write the site? Has anyone else said the same things anywhere else? Is there any way of checking this out? If the information is new, is there any proof?
6. When was the site produced? Is it up to date? Can you check to see if the information is up to date and not just the site?
7. Is the information biased in any way? Has the site got a particular reason for wanting you to think in a particular way? Is it a balanced view or does it only give one opinion?
8. Does the site tell you about choices open to you? Does the site give you advice? Does it tell you about other ideas?

### Net Scoring: Criteria to Assess the Quality of Health Internet Information

Net Scoring (Figure 11) is a French quality benchmarking system in use by many prominent French institutions, including the Centre Hospitalier Universitaire de Rouen, which developed CISMeF (Catalogue et Index des Sites Médicaux Francophones), the French equivalent of OMNI [27]. Net Scoring [28] provides a set of criteria for assessing the quality of health information on the Internet. To ensure objectivity in the development of these criteria, a diverse group of individuals, including representatives of professional organizations, medical doctors, engineers and lawyers, was gathered. Net Scoring comprises 49 criteria grouped into eight categories: credibility, content, links, design, interactivity, quantitative aspects, ethics and accessibility. Each criterion is assigned a weight according to its importance. An essential criterion is rated from 0 to 10. An important criterion is rated from 0 to 5, while a minor criterion is rated from 0 to 2. It is noteworthy that Net Scoring considers the "use of metadata" an essential criterion. The total of these weighted criteria gives the overall score of a site (the maximum score a resource can achieve is 297 points). Net Scoring criteria are presented in Appendix 1.

**Figure 11:** Net Scoring logo

### Medical Matrix

Medical Matrix (Figure 12) is a medical Web directory service that aims to improve the global medical community's access to useful digital clinical medicine resources [17] (registration is required but is free).

The Medical Matrix Project assigns star ranks to Internet resources based on their clinical utility as follows (Table 1):

**Table 1:** Medical Matrix Star Ranking System

*	Suitable, well-authored clinical content but lacking in substance, or currency. (1-10 points)
**	Clinical content is generally reliable and up-to-date. Site design is logical and easy to use. Limited usefulness as a regular clinical resource. (11-20 points)
***	Well-authored, accurate, current clinical content. Good site design, well-maintained and extensive functionality. Easily accessed and navigated by the routine user. An overall valuable clinical resource. (21-30 points)
****	Outstanding site across all categories and a premier web page for the discipline. (31-40 points)
*****	An award winning site for Medical Internet. (41-50 points)

Four main criteria are checked by the Medical Matrix Editorial Board when evaluating a resource:

- Dimension/usefulness for clinical applications: The resource enhances the knowledge base of the target clinician. Resource documents have current clinical relevance and importance, intellectual and scientific strength, and clarity of presentation. (1-20 points)
- Verifiability, clarity, and integrity: Resource document content is verifiable, endorsed, dated, current, and referenced. The material is original; the writing is clear; there is a minimization of bias; conclusions are reasonable and supported by evidence presented. The effort is ethical. The documents offer a comparison with relevant findings from other publications. Any conflict of interest is disclosed. (1-10 points)
- Evidence-based criteria: Conclusions are based on studies that are methodologically sound, meet statistical validity criteria and are clinically relevant. Conclusions are rated against a "gold standard" in that they are founded upon randomized trials with appropriate follow-up and are based on studies that make an independent, blind comparison of tests. (1-10 points)
- Media: Text, hypertext, or use of multimedia in the context of the resource. (1-10 points)
- Feel/ease of access: Easy to follow in terms of composition, presentation on the Web and integration within a larger database. (1-5 points)

**Figure 12:** Medical Matrix logo

### Principles Governing American Medical Association (AMA) Web Sites

**Figure 13:**
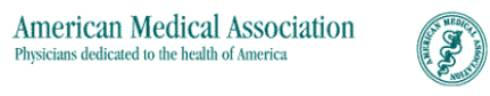
AMA logo

A standing committee composed of AMA staff members from the Scientific Publications and Multimedia, Publishing and Business Development, Ethical Standards, and Internet and Database Services areas (Figure 13) developed these guidelines, which were released in March 2000 [29]. The guidelines cover four areas, namely content, advertising and sponsorship, privacy and confidentiality, and e-commerce [29].

The principles for advertising and sponsorship cover many issues including guidance on the placement of digital advertisements. Just as a print advertisement would never be placed next to an editorial page on the same topic, a digital advertisement should never be adjacent to editorial content on the same topic. Readers should not be sent to a commercial site unless they choose to do so by clicking on an advertisement.

The principles for privacy and confidentiality aim at maintaining Web site visitors' rights to privacy and the confidentiality of personal information. Tracking of personal medical and health information, i.e., medical conditions, health-seeking behaviors and questions, and requests about drug therapies or medical devices or information pertaining to them, could breach an individual's personal privacy and reveal an individual's health data. Identifying details should be omitted if they are not essential, but patient data should never be altered or falsified in an attempt to attain anonymity. Information about individual medical conditions must not be collected without the express permission of the site visitor.

The principles for privacy and confidentiality also state that e-mail alerts and newsletters should only be sent upon a visitor's explicit request and should always contain an "unsubscribe" option.

### HSWG Criteria for Assessing the Quality of Health Information on the Internet

The Health Summit Working Group (HSWG), funded by the United States' Agency for Healthcare Research and Quality and the Health Information Technology Institute, selected, defined, ranked and evaluated seven major criteria for assessing the quality of Internet health information [30]. The credibility criterion covers the source, currency, relevance/utility, and editorial review process for the information. Resource content must be accurate (Table 2) and complete, and an appropriate disclaimer should be provided.

Disclosure includes informing the user of the purpose of the site, as well as any profiling or collection of information associated with using the site. Links are evaluated according to selection, architecture, content, and back linkages. Back linkages (also mentioned among Net Scoring criteria above) are links to one Web site from another. Many Web sites track and publish back linkages for the purpose of enhancing their credibility and marketability. The best way to evaluate back linkages is to examine the context in which they are used, that is, their purpose, relevance, credibility, and authority, as well as any associated bias. Design encompasses accessibility, logical organization (navigability), and internal search capability. Interactivity includes feedback mechanisms and means for exchange of information among users. The last criterion, caveats, involves clarification of whether the site function is to market products and services or is a primary information content provider.

**Table 2:** HSWG Hierarchy of Evidence

**Validity of Evidence**	**What to Look For**
++++ (Best Evidence)	Randomized controlled trials
+++	Non-randomized controlled trials
++	Well designed cohort or case-control analysis
+	Opinions of respected authorities, case reports, descriptive studies, reports of expert committees
No Evidence	Misrepresentation, fraud

### Silberg's Core Criteria for Measuring Quality (Caveat lector and viewor)

Silberg [31] suggested the following four core criteria for measuring the quality of medical Web sites:

- Authorship: The author(s) of a Web page, along with their affiliations and credentials, should be clearly stated. Ideally, there should be the facility to contact the author(s) by e-mail.
- Attribution: If a Web site is quoting research or evidence then the source of this data must be explicitly stated.
- Disclosure: The owner of the Web site must be prominently displayed, along with any sponsorship or advertising deals that could constitute a potential conflict of interest.
- Currency: Web pages should indicate when the page was created, and when it was last updated.

The authors have noted that some webmasters use JavaScript within their pages to pick up the client machine's current date and display it after a "last updated" or similar string, fooling the reader into thinking that these pages were updated on the same day (i.e., a false impression of currency).

### Price's PILOT Method for Evaluating Medical Web Sites

The PILOT method for evaluating medical Web sites consists of five criteria (Table 3) [32]:

**Table 3:** Price's Pilot Method

**Purpose:**	If the site has a mission statement, read it. If not, read the home page and analyse the site's purpose. Does it inform and educate? Or is designed to persuade, sell, outrage or entertain?
**Information:**	Truly useful medical Web sites offer valuable information and emphasise facts rather than opinion and testimonials. If the site is selling anything, ask yourself if that influences the content.
**Links:**	The best sites want to inform you and are happy to recommend additional Web sites to further your knowledge in that topic or related topics. The best links are rated or reviewed.
**Originator:**	Who is responsible for the information? Best bets for sound medical information are medical societies, consumer-advocacy groups, well-known hospitals, and government- and university-sponsored sites.
**Timeliness:**	Medical information is only useful if it is current. Look for sites that update frequently.

### Developing a Quality Benchmarking System for the NeLH

According to the Centre for Health Information Quality (C-H-i-Q) [25], a new discipline will probably emerge in the near future, with the development of an academic qualification in health information appraisal. Healthcare professionals trained in this domain will be the ideal NeLH "knowledge miners" or "quality assurance officers." We believe that a quality evaluation software wizard should be developed, possibly by pooling the best criteria and methods from all the quality benchmarking systems that are available today to produce an all-in knowledge-hallmarking instrument. The aim of this wizard will be to assist (not to replace) NeLH knowledge miners in their work and ensure that they consistently and explicitly follow all the required evaluation steps. This wizard should be also able to write evaluation results and details of the resource being evaluated (e.g. URL, score or rating, currency information, etc.) and any other relevant meta-information to the appropriate NeLH page or to the record pointing to this resource in the NeLH resource catalogue or database. To develop a good quality benchmarking system, a standard statement must first be defined. The standard statement is the hub on which the other elements of the standard revolve. It describes an agreed level of quality appropriate to the organizational and target readers needs. It specifies a desirable, acceptable and achievable quality level. The next step is to develop criteria that clearly and precisely specify the quality level that must be present in order to satisfy the standard. For any given quality standard, it is possible to write a great many criteria. The quality assurance team responsible for the development of the quality benchmarking system, therefore, has to choose criteria on the basis of a sound principle like AMOUR [33]. AMOUR defines a set of five clear questions/goals that each of the candidate criteria must answer:

- is the criterion Achievable? The development group must choose between idealism (unattainable standards) and realism.
- is it Measurable? A standard statement might not be worded in measurable terms but a criterion must be. When writing a criterion it is helpful to consider "how can a check be made that a given resource fulfils the criterion?" and find a clear and practical answer to this question.
- is it Observable? For a phenomenon to be observable it must be detectable through the senses. If a criterion refers to unobservable (very abstract) objective, it will be almost impossible to determine whether or not this objective has been satisfied by any given resource under examination. A criterion which uses vague terms such as "appropriate content" or "information should not be biased" is very difficult to use (and very subjective).
- is it Understandable? The development team should unambiguously and explicitly define the meanings of linguistic variables like "appropriate" or "suitable." The rule is always to be clear, objective and specific.
- is it Reasonable? All involved or targeted "players" (information providers, patients and professionals) must be represented within the development group formulating the benchmarking system to ensure that all pertinent players find the final criteria reasonable.

### Selection and Evaluation of Candidate Web Resources for the Dermatology VBL

The authors used Web Inspector, an offline metasearch tool [34], some specialized medical search engines and directories, e.g. HON MedHunt and HONselect [9], plus the personal knowledge of the first author (who is a dermatologist) about good dermatology Web resources to locate candidate sites.

Six major Internet search engines (Euroseek, Excite, Infoseek, Yahoo, All the Web and Google) were queried at the same time by Web Inspector (on 13 May 2000) using the keyword 'psoriasis'. The maximum number of results per search engine was set to 300. After the automatic removal by Web Inspector of all duplicate and dead links, 413 documents (containing the word 'psoriasis') remained for manual exploration by the authors.

We tried to include a balanced mix of resources that can be used to answer a variety of information queries and needs. The selected resources (Appendix 2) include sites intended mainly for patients, as well as sites targeting healthcare professionals. In addition to psoriasis-specific resources, the authors have also compiled a list of some of the very useful general dermatology resources on the Web for patients and healthcare professionals. These "general" resources can support continuing professional education and also provide different types of information on the rest of skin diseases.

Sites that are purely textual, searchable databases, atlases (dermatology image banks), sites that mix text, graphics and interactive elements (e.g. educational simulations) were all considered for inclusion, as long as they did not rely on sophisticated browser plug-ins and/or non-standard Web technologies to display their information. JavaScript (when properly implemented), Adobe Acrobat Reader and RealNetworks RealPlayer are examples of respectable, mainstream Web technologies and browser plug-ins or extensions. They are freely available, very stable, lightweight and supported on a wide variety of platforms, and therefore pages using them were not excluded. Web directories that are just link lists (pointers to other resources) were excluded unless they constituted a real resource guide offering additional evaluative or descriptive information. Information providers whose contributions to the knowledge base of dermatology have long been recognized as authoritative, e.g. the British Association of Dermatologists (http://www.bad.org.uk/ [accessed 27 August 2000], were preferred.

Sites displaying advertisements or sponsored by commercial bodies, e.g. sites endorsed by pharmaceutical companies, were typically excluded for obvious reasons, unless the author felt there were no real conflicts of interest, possibility of bias or any other hidden or malicious motives. For example, the author has included the New Zealand DermNet (http://www.dermnet.org.nz [accessed 27 August 2000], which remains a very good and unbiased resource, despite the fact that it displays pharmaceutical advertisements.

Sites that are not regularly updated were excluded, based on the concept that every piece of information should have an "expiry date" after which it should be revised and, if necessary, updated. But again exceptions to this rule had to be made to include some sites with inadequate currency information, but providing very good basic information that is less likely to change over time.

Deleting the right hand side of a resource URL in steps has proved very helpful in providing additional insight and information concerning the credibility of the authors and publishers of some resources, e.g. http://www.ee.oulu.fi/~juko/psoriasis.html (the resource), http://www.ee.oulu.fi/~juko/ (the author) and http://www.ee.oulu.fi/ (the author's the academic institution).

## Results

**Figure 14:**
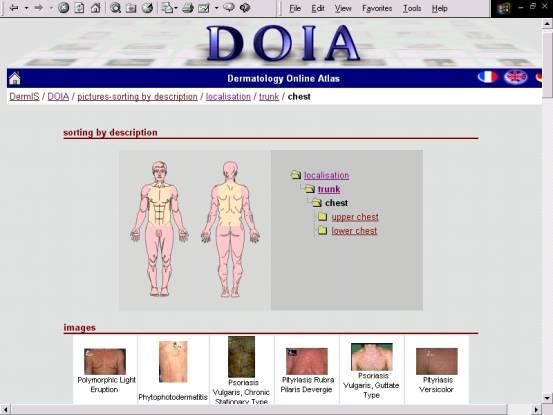


Over 90 professional and patient-oriented Web resources on psoriasis and dermatology in general have been hand-selected for possible inclusion in the forthcoming Dermatology VBL (Appendix 2). Among the selected electronic Web-based atlases is Dermatology Online Atlas (DOIA) at the University of Erlangen, Germany (Figure 14) [35]. The history of this Atlas of Dermatology goes back to 1994, the early days of the World Wide Web. In these days, a German research project was launched to use this new technology for the benefit of medical education and teaching, especially in dermatology.

This online atlas of dermatology surpasses any printed image atlas and integrates both interactive and dynamic components, with more than 3000 high quality images of more than 600 dermatological diagnoses and differential diagnoses. Morphological features of the images have been described by dermatologists. The site offers a database query interface for advanced image selection by different criteria, including localization, morphological features, age and gender. With each image or set of images covering the same diagnosis, direct links are provided to other images of suitable differential diagnoses within the atlas. Also provided are commented and rated links to external Web sites offering further information on the respective dermatological disease. Special links allow direct, fast and easy access to disease-related information in databases like MEDLINE and OMIM (Online Mendelian Inheritance in Man). The atlas can be also browsed in 'quizmode' for training [36]. While the authors did their best to observe the essence and spirit of all the quality benchmarking systems described above during the selection and evaluation process, the list of candidate resources (Appendix 2) should not be considered an exhaustive and final selection, but rather a first-pass filtering. The identified candidate sites should be subjected to more scrutiny to determine their final status, whether suitable or not for inclusion in the NeLH. Quality benchmarking of health-related resources will always depend on a human assessor, and as such, it will always have attached to it an inevitable element of subjectivity that cannot be corrected by simply providing numerical scales for rating a resource. These numerical scales can only offer apparent, but not true objectivity. According to Delamothe [37], rating the quality of medical Web sites may be impossible. One option he presents is to rate the process by which the content was produced rather than the content itself, i.e., rate the assessor or information provider rather than individual resources. Thus a medical journal's Web site containing peer-reviewed material would rate higher than a commercial site selling miracle cures for cancer.

## Discussion

Online (e.g. http://www.metacrawler.com/ [accessed 27 August 2000] and offline (e.g. Web Inspector [34]) metasearch Web tools can greatly assist human NeLH knowledge miners by performing an automatic preliminary mining and filtering of Web pages on a given topic, thus facilitating the final filtering task of the human knowledge miner. By querying multiple search engines at the same time the potential for finding information is much greater. Moreover, users are spared the time spent manually visiting a number of search engines and re-keying their query or in following dead or duplicate links. However, the NeLH should always remain a human-maintained catalogue with value added contents. Skilled, methodical, organized human reviewing, selection and filtering based on well-defined quality appraisal criteria seems likely to be the key ingredient in the envisaged NeLH service. Proper training of human assessors to develop good discernment skills is as important as the quality benchmarking checklists themselves. The authors recommend that assessors take the "Internet Detective" interactive tutorial on evaluating the quality of Internet resources [38]. This tutorial is provided by the Social Science Information Gateway (SOSIG), which is part of the UK Resource Discovery Network. In the near future, MedCERTAIN [13] might become an international (or at least European) standard for rating health-related Web sites if it succeeds in setting the norm for quality benchmarking criteria and labeling infrastructure. By promoting the application of agreed quality guidelines and codes of ethics by all health information providers and not just within the NeLH, the overall quality of the Web should improve with time. This improvement in quality will always be the result of a collaborative world-wide effort based on the goodwill of "informed net citizens" and "professional obligation" of healthcare professionals and information providers, rather than on any enforced laws (i.e., self-regulation). Ultimately, the Web will become a reliable and integral part of the care space.

## Abbreviations

AGEC: Advisory Group on Evaluation Criteria
C-H-i-Q: Centre for Health Information Quality
NeLH: National electronic Library for Health
NHS: National Health Service
NICE: National Institute for Clinical Excellence
RDF: Resource Description Framework
UMLS: Unified Medical Language System
VBL: Virtual Branch Libraries
vortals: Vertical portal targets a specific topic
XML: eXtensible Markup Language

## Appendix 1

### Net Scoring Criteria to Assess the Quality of Health Information

**Table 4:** Net Scoring Criteria to Assess the Quality of Health Information

**1 Credibility** **(100 points)**	1.1 Source 1.1a Name, logo and references of the institution on each document of the site (essential criterion) 1.1b Name and title of author on each document of the site (essential criterion) 1.2 Disclosure 1.2a Context: source of financing, independence of the author(s) (essential criterion) 1.2b Conflict of interest (important criterion) 1.2c Influence, bias (important criterion) 1.3 Updating: currency information of the site including date of creation, date of last update and eventually date of last version (essential criterion) 1.4 Relevance/utility (essential criterion) 1.5 Editorial review process (essential criterion) 1.5a Webmastering process (important criterion) 1.5b Scientific review process (important criterion) 1.6. Target/purpose of the web site; access to the site (free or not, reserved or not) (important criterion) 1.7. Quality of the language and/or translation (important criterion) 1.8 Use of metadata (essential criterion)
**2 Content** **(79 points)**	2.1 Accuracy (essential criterion) 2.2 Hierarchy of evidence (important criterion) 2.3 Original Source Stated (essential criterion) 2.4 Disclaimer (important criterion) 2.5 Logic organization (navigability) (essential criterion) 2.5a Quality of the internal search engine (important criterion) 2.5b General index (important criterion) 2.5c What's new page (important criterion) 2.5d Help page (minor criterion) 2.5e Map of the site (minor criterion) 2.7 Omissions noted (essential criterion) 2.8 Fast load of the site and its different pages (important criterion) 2.9 Clear display of available information categories (factual data, abstracts, full-text documents, catalogue, databases) (important criterion)
**3 Hyperlinks** **(52 points)**	3.1 Selection (essential criterion) 3.2 Architecture (important criterion) 3.3 Content (essential criterion) 3.4 Back-links or number of hyperlinks to the resource in question (Web Impact Factor) (important criterion) 3.5 Regular verification that hyper-links are functioning, i.e., no broken links (important criterion) 3.6 In case of modification of the site structure, link between old and new HTML documents (important criterion) 3.7 Distinction between internal and external hyper-links (minor criterion)
**4 Design** **(20 points)**	4.1 Design of the site (essential criterion) 4.2 Readability of the text, images, or video (important criterion) 4.3 Quality of the print (important criterion)
**5 Interactivity** **(17 points)**	5.1 Mechanism for feedback: Email of the author on every document (essential criterion) 5.2 Forums, chat (minor criterion) 5.3 Users must be clearly informed if the site is using cookies or any similar technologies to trace their logging into the site and to retrieve information about their machines (even if the tracing is done in anonymous form). Users must be able to opt in or opt out of functions that track personal or machine information at any time (important criterion)
**6 Quantitative aspects** **(9 points)**	6.1 Number of machines (IP addresses) visiting the site and number of visualised documents (important criterion) 6.2 Number of press releases (minor criterion) 6.3 Number of scientific production from the site, including bibliometric criteria (minor criterion)
**7 Ethics** **(20 points)**	7.1 Liability of the reader (essential criterion) 7.2 Medical privacy (essential criterion)
**8 Accessibility** **(10 points)**	8.1 Accessibility from the main search engines and catalogues (important criterion) 8.2 Intuitive address of a site (important criterion)
	**297 points maximum**

## Appendix 2

### Patient Information

**Table 5:** List of candidate general dermatology and psoriasis Web resources for inclusion in the NeLH Dermatology VBL. Please note that URLs do not contain white spaces or line breaks

**General Resources**	
**Site**	**URL** (all URLs have been accessed 27 August 2000)
NHS Direct (U.K.) [26]	http://www.nhsdirect.nhs.uk/
U.K. Dermatology Patient Support Groups (U.K.)	http://www.kcl.ac.uk/depsta/medicine/dermatology/other/ukgroup.html
New Zealand DermNet for Patients	http://www.dermnet.org.nz/contents.a.html
American Academy of Dermatology Patient Information	http://www.aad.org/pamphlets/index.html
American Academy of Family Physicians - Patient Information (Skin and Hair)	http://www.aafp.org/patientinfo/body13.html
American Academy of Family Physicians - Patient Information (Skin Disorders)	http://www.aafp.org/patientinfo/common9.html
Educational Information on Common Skin Diseases for Patients - National Skin Centre, Singapore	http://www.nsc.gov.sg/commskin/skin.html
**Psoriasis: Online Patient Information and Pamphlets**	
NHS Direct Audio clip on psoriasis from the College of Health Healthline Tapes service (requires RealPlayer - www.real.com)	http://www.nhsdirect.nhs.uk/audio_cache/53.ram
PPP healthcare International-Health Info-psoriasis (U.K.)	http://www.ppphealthcare.co.uk/html/health/psors.htm
NIH - Questions and Answers About Psoriasis (U.S.A.)	http://www.nih.gov/niams/healthinfo/psoriasis.htm
MEDLINEplus - Psoriasis (U.S.A.)	http://www.nlm.nih.gov/medlineplus/psoriasis.html
AAD Psoriasis Pamphlet (U.S.A.)	http://www.aad.org/pamphlets/Psoriasis.html
American Academy of Family Physicians: What Can Be Done for Psoriasis (U.S.A.)	http://www.aafp.org/patientinfo/psoriasi.html
Psoriasis at Medinfo (U.K.)	http://www.medinfo.co.uk/conditions/psoriasis.html
MCW HealthLink: Psoriasis (U.S.A.)	http://healthlink.mcw.edu/article/926054522.html
Psoriasis Research Institute: Facts about Psoriasis (U.S.A.)	http://www.psoriasis-help.org/facts.html
Psoriatic Arthritis at the University of Washington (U.S.A.)	http://www.orthop.washington.edu/bonejoint/wzzzzzzz1_2.html
**Psoriasis: Online Patient Information and Pamphlets**	
**Site**	**URL** (all URLs have been accessed 27 August 2000)
DermWeb Handout: Psoriasis: What is it and How is it Treated? (Canada)	http://www.derm.ubc.ca/skincare/psoriasis/psorhand.html
National Skin Centre: Psoriasis (Singapore)	http://www.nsc.gov.sg/commskin/Psoriasi/psoriasi.html
Beware of Web quackery and the so-called miraculous cures: Psoriasis Hall of PShame (U.S.A.)	http://www.pinch.com/skin/pshame.html
**Psoriasis: Online Organizations**	
The Psoriatic Arthropathy Alliance (U.K.)	http://www.paalliance.org/
National Psoriasis Foundation (U.S.A.)	http://www.psoriasis.org/
Canadian Psoriasis Foundation	http://www.psoriasis.ca/
Psoriasis Society of Canada	http://www.psoriasissociety.org/
**Health Care Professionals**	
**General Resources/Research Tools**	
Cochrane Skin Group	http://www.nottingham.ac.uk/~muzd/
The British Association of Dermatologists	http://www.bad.org.uk/
New Zealand DermNet for Dermatologists	http://www.dermnet.org.nz/contents.b.html
New Zealand DermNet for General Practitioners	http://www.dermnet.org.nz/contents.c.html
Skindex - Dynamic Dermatology (Dr. Thomas B. Fitzpatrick - U.S.A.)	http://www.skindex.com/
Skin Diseases - Primary Care Clinical Practice Guidelines, University of California, San Francisco (U.S.A.)	http://medicine.ucsf.edu/resources/guidelines/guide12.html
Archives of Dermatology (Full text Journal - U.S.A.)	http://archderm.ama-assn.org/
myMedline.com Dermatology Medline Engine	http://www.medplaza.com/medline/derm.php3
Hardin Library for the Health Sciences, University of Iowa - PubMed Medline search Dermatology & Skin Diseases (U.S.A.)	http://www.lib.uiowa.edu/hardin/pm/derm.html
Health On the Net Foundation: HONselect - Skin and Connective Tissue Diseases (Switzerland)	http://www.hon.ch/cgi-bin/HONselect?browse+C17
NIH Library - Skin Diseases Sites (U.S.A.)	http://libwww.ncrr.nih.gov/internet/Biomedicine_Science/bsskincon.htm
**General Resources/Research Tools**	
**Site**	**URL** (all URLs have been accessed 27 August 2000)
Department of Dermatology Links Page at the University of Wales College of Medicine, Cardiff (U.K.)	http://www.uwcm.ac.uk/uwcm/dm/dermlink.html
Matrix Dermatology Resources (U.S.A.)	http://matrix.ucdavis.edu/
DermWeb, the Home Page for the Division of Dermatology at the University of British Columbia (Canada)	http://www.dermatology.org/
Dermatology Medical Journals - WebMedLit	http://webmedlit.silverplatter.com/topics/derm.html
Medical Matrix: Dermatology (Requires registration; registration is free)	http://www.medmatrix.org/_SPages/Dermatology.asp
Skin and Connective Tissue Diseases - MeSH classified index, Karolinska Institute (Sweden)	http://www.mic.ki.se/Diseases/c17.html
Dermatonet for Professionals (France)	http://www.dermatonet.com/mede.htm
**Psoriasis: Comprehensive Reviews**	
eMedicine Online Text: Psoriasis (U.S.A.)	http://emedicine.com/cgi-bin/foxweb.exe/showsection@d:/em/ga?book=emerg&topicid=489
The Merck Manual of Diagnosis and Therapy: Psoriasis (U.S.A.)	http://www.merck.com/pubs/mmanual/section10/chapter117/117b.htm
Psoriasis in Handbook of Dermatology & Venereology, 2nd Edition, Social Hygiene Service, Department of Health, Hong Kong	http://www.hkmj.org.hk/skin/psoriasi.htm
**Psoriasis: Genetics**	
Psoriasis Genetics Laboratory, University of Michigan (U.S.A.)	http://www.psoriasis.umich.edu/index.html
OMIM Psoriasis Inheritance and Genetics Research (U.S.A.)	http://www.ncbi.nlm.nih.gov/htbin-post/Omim/getmim?search=psoriasis
**Psoriasis: Photographs**	
Dermatology Online Atlas (DOIA) Erlangen: Psoriasis (Germany)	http://www.dermis.net/cgi-bin/dbquery/frames.asp?diag=psoriasis&lokalisation=&zusatz=&eff1=&eff2=&farbe=&weitere=&age1=&age2=&lang=e&nr=0
Psoriasis of the nails	http://www.nh.ultranet.com/~mhabif/html/psoriasis.html
**Psoriasis: Research/Online Pre-formulated Queries**	
eBMJ - Psoriasis Articles (U.K.)	http://www.bmj.com/cgi/search?hits=100&fulltext=psoriasis
Honselect - Psoriasis (Switzerland)	http://www.hon.ch/cgi-bin/HONselect?browse+C17.800.859.675
**Psoriasis: Research/Online Pre-formulated Queries**	
**Site**	**URL** (all URLs have been accessed 27 August 2000)
CliniWeb Search Results for Psoriasis (U.S.A.)	http://www.ohsu.edu/cliniweb/C17/C17.800.859.html#C17.800.859.675
Medical World Search Results for Psoriasis (U.S.A.)	http://www.mwsearch.com/cgi-bin/new_search.pl?search_target=Major+Sites&initial_query=psoriasis&start_docid=1&which_form=initial_search
Archives of Dermatology Search Results for Psoriasis (free full text articles available) (U.S.A.)	http://pubsearch.ama-assn.org/search?action=filtersearch&QueryZip=psoriasis&ResultMaxDocs=100000&Filter=search%5Ffilter%2Ehts&ResultTemplate=search%5Fresults%2Ehts&Collection=DERM&SortSpec=collPriority+desc&ResultStart=1&ResultCount=10&SF=Score&SO=desc&FreeText=psoriasis&volume_query=&title_query=&author_query=&startpage_query=&affil_query=&f_date=&f_fmonth=&f_fyear=&f_tmonth=&f_tyear=
NIH MEDLINE abstracts on psoriasis (All) (U.S.A.)	http://www.ncbi.nlm.nih.gov/htbin-post/Entrez/query?db=m&form=4&term=Psoriasis[MAJR]+AND+Human[MESH]+AND+English[LANG]&dopt=d&relpubdate=1+Year&dispmax=20
NIH MEDLINE abstracts on psoriasis (Review) (U.S.A.)	http://www.ncbi.nlm.nih.gov/htbin-post/Entrez/query?db=m&form=4&term=Psoriasis[MAJR]+AND+Human[MESH]+AND+English[LANG]+AND+review[PTYP]&dopt=d&relpubdate=1+Year&dispmax=20
NIH MEDLINE abstracts on psoriasis (Diagnosis) (U.S.A.)	http://www.ncbi.nlm.nih.gov/htbin-post/Entrez/query?db=m&form=4&term=Psoriasis[MAJR]+AND+Human[MESH]+AND+English[LANG]+AND+(sensitivity+and+specificity[MESH]+OR+sensitivity[WORD]OR+diagnosis[SH]+OR+diagnostic+use[SH]+OR+specificity[WORD])&dopt=d&relpubdate=1+Year&dispmax=20
NIH - Psoriasis info (U.S.A.)	http://search.info.nih.gov/s97is.vts?Collection=NIH&Summary=1&ResultTemplate=NIH.hts&queryText=psoriasis
Computer Image Analysing (CIA) System for Psoriasis Area Assessment (Research - Medical Informatics/Machine Vision - Finland)	http://www.ee.oulu.fi/~juko/psoriasis.html
**Psoriasis: Treatment**	
Psoriasis treatments [Bandolier - May 1998; 51-5] (U.K.)	http://www.jr2.ox.ac.uk/bandolier/band51/b51-5.html
Topical capsaicin in Psoriasis [Bandolier - Jul 1996; 29-6] (U.K.)	http://www.jr2.ox.ac.uk/bandolier/band29/b29-6.html
Cochrane - Interventions for guttate psoriasis (U.K.)	http://www.update-software.com/ccweb/cochrane/revabstr/ab001213.htm
Cochrane - Antistreptococcal interventions for guttate and chronic plaque psoriasis (U.K.)	http://www.update-software.com/ccweb/cochrane/revabstr/ab001976.htm
Cochrane - Interventions for psoriatic arthritis (U.K.)	http://www.update-software.com/ccweb/cochrane/revabstr/ab000212.htm
American Academy of dermatology Guidelines of Care for Psoriasis (U.S.A.)	http://www.aad.org/Guidelines/psoriasis.html
American Academy of Family Physicians: Topical Psoriasis Therapy (U.S.A.)	http://www.aafp.org/afp/990215ap/957.html
Dermatology Times - Psoriasis Treatments (U.S.A.)	http://www.dermatologytimes.com/psorias.html
For Nurses: Psoriasis: what to tell your patients (NurseWeek, U.S.A.)	http://www.nurseweek.com/ce/ce804a.html
NIH MEDLINE abstracts on psoriasis (Treatment Research - U.S.A.)	http://www.pinch.com/hit?medline=psoriasis+%5Bti%5D+AND+(treat*+OR+therap*)
**Professional Education**	
**Online Textbooks, Lectures and Clinical Case Simulations**	
eMedicine Online Text - Dermatology (U.S.A.)	http://www.emedicine.com/derm/index.shtml
Handbook of Dermatology & Venereology, 2nd Edition, Social Hygiene Service, Department of Health, Hong Kong	http://www.hkmj.org.hk/skin/
The Electronic Textbook of Dermatology (U.S.A.)	http://telemedicine.org/stamford.htm
Interactive Dermatology Cases, UCLA Medical School (U.S.A.)	http://idtu.medsch.ucla.edu/scripts/Tangocgi.exe/Brian/derm4.qry
Simulated Dermatology Patient (Taiwan)	http://ades.tmc.edu.tw/english/pcare/simulation/default.htm
Multimedia Dermatology Course (Taiwan)	http://ades.tmc.edu.tw/english/pcare/course/default.htm
Dermatology and Dermatopathology Online Lectures, University of Rochester Medical Centre (U.S.A.)	http://www.urmc.rochester.edu/derm/lectures.html
Dermatology for Students (Skin Tumours and Other Skin Diseases), University of California Davis (U.S.A.)	http://matrix.ucdavis.edu/tumors.html
Developmental Biology of the Skin: An Interactive Tutorial, University of Washington (U.S.A.)	http://www.hslib.washington.edu/courses/hubio567/
**Online Atlases/Image Banks**	
DermIS - DOIA Dermatology Online Atlas - English version. (Germany)	http://dermis.net/bilddb/index_e.htm
Dermatology Atlas - Loyola University Dermatology Medical Education Website (U.S.A.)	http://www.meddean.luc.edu/lumen/meded/medicine/dermatology/melton/atlas.htm
Dermatology Images, University of Kansas Medical Center (U.S.A.)	http://www.kumc.edu/instruction/medicine/cont-ed/infotech/der-main.htm
Skin Images, Harvard Medical School (U.S.A.)	http://hms.medweb.harvard.edu/nmw/qry/imgpages/imgcollection.cfm?set=HS_Skin
Dermatology Images, University of Iowa (U.S.A.)	http://tray.dermatology.uiowa.edu/DermImag.htm (accessed 12 June 2000)
Dermatology Image Bank at the University of Utah School of Medicine (U.S.A.)	http://www-medlib.med.utah.edu/kw/derm/
Dermatopathology Atlas, Indiana University (U.S.A.)	http://www.pathology.iupui.edu/derm.html
Dermatology and Rheumatology Online Atlas (Italy)	http://www.archrheumatol.net/atlas/index-page.htm
Anatomy of the skin (Electron Microscopy), Department of Dermatology, Mie University School Of Medicine (Japan)	http://www.medic.mie-u.ac.jp/derma/anatomy.html
**Evidence-based Practice**	
Bigby M. Welcome to evidence-based medicine. Arch Dermatol 1998; 134:1516	http://archderm.ama-assn.org/issues/v134n12/full/ded8023.html
Sharon E. Straus SE and Sackett DL. Bringing evidence to the clinic. Arch Dermatol 1998; 134:1519	http://archderm.ama-assn.org/issues/v134n12/full/ded8017.html
Bigby M. Evidence-based medicine in a nutshell: a guide to finding and using the best evidence in caring for patients. Arch Dermatol 1998; 134:1609	http://archderm.ama-assn.org/issues/v134n12/full/dre8017.html
Williams H. Adetugbo K, Li Wan Po A, Naldi L, Diepgen T and Murrell D. The Cochrane Skin Group: preparing, maintaining, and disseminating systematic reviews of clinical interventions in dermatology. Arch Dermatol 1998; 134:1620	http://archderm.ama-assn.org/issues/v134n12/full/dre8023.html
Adetugbo K and Williams H. How well are randomized controlled trials reported in the dermatology literature? Arch Dermatol 2000; 136:381	http://archderm.ama-assn.org/issues/v136n3/full/doc9010.html
The Outputs database of the R&D Programme (DoH - U.K.)	http://www.doh.gov.uk/research/index.htm
Primary Care Clinical Practice Guideline, University of California, San Francisco (U.S.A.)	http://medicine.ucsf.edu/resources/guidelines/intro.html
